# Mineral Solubilizing Rhizobacterial Strains Mediated Biostimulation of Rhodes Grass Seedlings

**DOI:** 10.3390/microorganisms11102543

**Published:** 2023-10-12

**Authors:** Shaista Javaid, Saira Mushtaq, Muhammad Zahid Mumtaz, Ghulam Rasool, Tahir Naqqash, Maha Afzal, Uzma Mushtaq, Hayssam M. Ali, Muhammad Fakhar-U-Zaman Akhtar, Ghulam Abbas, Lingling Li

**Affiliations:** 1 Institute of Molecular Biology and Biotechnology, The University of Lahore Main Campus, Lahore 54000, Pakistan; 2 Institute of Molecular Biology and Biotechnology, Bahauddin Zakariya University, Multan 60800, Pakistan; 3 Department of Botany and Microbiology, College of Science, King Saud University, Riyadh 11451, Saudi Arabia; hayhassan@ksu.edu.sa; 4 Department of Soil Science, The Islamia University of Bahawalpur, Bahawalpur 63100, Pakistan; 5 Centre for Climate Research and Development, COMSATS University Islamabad, Islamabad 45550, Pakistan; 6 College of Agronomy, Gansu Agricultural University, Lanzhou 730070, China; lill@gsau.edu.cn; 7 State Key Laboratory of Aridland Crop Science, Gansu Agricultural University, Lanzhou 730070, China

**Keywords:** *Bacillus*, *Chloris gayana*, enzymatic activities, mineral solubilization, *Staphylococcus*, partial gene sequencing

## Abstract

Minerals play a dynamic role in plant growth and development. However, most of these mineral nutrients are unavailable to plants due to their presence in fixed forms, which causes significant losses in crop production. An effective strategy to overcome this challenge is using mineral solubilizing bacteria, which can convert insoluble forms of minerals into soluble ones that plants can quickly assimilate, thus enhancing their availability in nutrient-depleted soils. The main objective of the present study was to isolate and characterize mineral solubilizing rhizobacteria and to assess their plant growth-promoting potential for Rhodes grass. Twenty-five rhizobacterial strains were isolated on a nutrient agar medium. They were characterized for solubilization of insoluble minerals (phosphate, potassium, zinc, and manganese), indole acetic acid production, enzymatic activities, and various morphological traits. The selected strains were also evaluated for their potential to promote the growth of Rhodes grass seedlings. Among tested strains, eight strains demonstrated strong qualitative and quantitative solubilization of insoluble phosphate. Strain MS2 reported the highest phosphate solubilization index, phosphate solubilization efficiency, available phosphorus concentration, and reduction in medium pH. Among tested strains, 75% were positive for zinc and manganese solubilization, and 37.5% were positive for potassium solubilization. Strain MS2 demonstrated the highest quantitative manganese solubilization, while strains MS7 and SM4 reported the highest solubilization of zinc and potassium through acidifying their respective media. The strain SM4 demonstrated the most increased IAA production in the presence and absence of L-tryptophan. The majority of strains were positive for various enzymes, including urease, catalase protease, and amylase activities. However, these strains were negative for coagulase activity except strains SM7 and MS7. Based on 16S rRNA gene sequencing, six strains, namely, SM2, SM4, SM5, MS1, MS2, and MS4, were identified as *Bacillus cereus*, while strains SM7 and MS7 were identified as *Staphylococcus saprophyticus* and *Staphylococcus haemolyticus*. These strains significantly improved growth attributes of Rhodes grass, such as root length, shoot length, and root and shoot fresh and dry biomasses compared to the uninoculated control group. The present study highlights the significance of mineral solubilizing and enzyme-producing rhizobacterial strains as potential bioinoculants to enhance Rhodes grass growth under mineral-deficient conditions sustainably.

## 1. Introduction

Rhodes grass (*Chloris gayana* Kunth), native to Africa [1,2], was named after the well-known Cecil Rhodes, who popularized its use. Its role is prominent in forage crops and is known for its versatility, adaptability to diverse environments and soil types, and remarkable drought resistance [3,4]. Furthermore, Rhodes grass boasts a high nutritional value, with a protein content ranging from 9% to 12%, rendering it a valuable feed source for livestock, particularly in tropical and subtropical regions [4,5]. Moreover, Rhodes grass is helpful for the rotation of grasslands and effective for soil conservation due to its extensive root system and ability to thrive in degraded soils. The hay form of Rhodes grass is much more prevalent around the globe, notably in Gulf countries such as UAE, Qatar, and Saudi Arabia [5]. In Pakistan, fodder production is a crucial energy source to feed the livestock during the lean time. According to previous research, large-scale cultivation of Rhodes grass started in 2008. Over 100,000 acres of Balochistan, Sindh, and Punjab are dedicated to cultivating Rhodes grass for fodder production [6,7]. Improved forage grasses are one of the primary feed sources for grazing animals. Therefore, to address the feed shortage and increase livestock production, it is crucial to enhance the production of high-quality forages with greater biomass [7].

Chemical fertilizers are utilized to sustain soil fertility, but the soil cannot hold the applied amount of fertilizer. The excessive and long-term use of synthetic fertilizers has undesirable impacts on soil, environment, plants, and human health [8]. A significant proportion of the applied fertilizer is transformed into insoluble forms and fixed in the soil [9]. There is a need to improve nutrient use efficiency in the crops in an economically reliable and ecologically safer manner [10]. Applying effective microbial bioinoculants as plant growth-promoting (PGP) agents is an alternative and promising biotechnological strategy against synthetic chemicals [11]. The bacterial bioinoculants consist of beneficial plant growth-promoting rhizobacteria (PGPR) that reside in the rhizosphere in association with roots to improve plant growth and development [12]. Microbial bioinoculants are safer, cost-effective, and eco-friendly than agrochemicals and can improve soil structure and fertility [13]. 

Mineral solubilizing bacteria (MSB) have the potential to solubilize insoluble mineral forms such as phosphate (P), potassium (K), and zinc (Zn) that can be absorbed by plants [14,15]. Among MSB, P-solubilizing bacteria were reported to solubilize various inorganic forms of insoluble minerals, including tricalcium phosphate, fluoroapatite, francolites, hydroxyapatite, augellite, barrandite, crandallite, strengite, variscite, wavellite [16]. K-solubilizing bacteria were involved in solubilizing insoluble minerals, including micas, muscovite, feldspar, biotite, illite, and orthoclase [17]. The insoluble Zn minerals, including zinc oxide, zincite, zinc silicates, willemite, sphalerite, smithsonite, and zinc sulfide, were reported to be solubilized by Zn solubilizing bacteria [18]. These MSBs adopted various mechanisms in nature to give plants nutrients, such as mineralization, solubilization, and mobilization [19]. Among these mechanisms, solubilization of minerals converts insoluble form into soluble form by producing organic acid secretion, chelation, acidification, exchange reaction, and secretion of protons [20,21]. Most effective MSBs include bacterial genera such as *Azospirillum, Acinetobacter, Azotobacter, Arthobacter, Burkholderia, Bacillus, Erwinia, Enterobacter, Pseudomonas, Rhizobium, Rhodococcus, Thiobacillus, Klebsiella, Frankia,* and *Serratia* [22,23,24,25,26,27].

Thus, present study aimed (i) to isolate potential plant growth-promoting rhizobacterial strains from the rhizosphere soil of Rhodes grass, (ii) to screen them for in vitro solubilization of insoluble minerals, (iii) in vitro screening of MSB strains for PGP attributes (iv) to identify the promising minerals solubilizing PGPR through 16S rRNA gene sequencing, and (v) to evaluate the impact of mineral solubilizing bacterial strains inoculation on Rhodes grass growth parameters and nutrients uptake under presence of insoluble mineral compounds.

## 2. Materials and Methods

### 2.1. Isolation of Rhizobacteria

Rhizosphere soil samples of Rhodes grass were collected from two different sites: site-I Rahim Yar Khan, Punjab, Pakistan (located at Latitude 28.49 N, Longitude 70.25 E, and elevation 83.0 m); and site-II Ghotki, Sindh, Pakistan (located at Latitude 27.96 N, Longitude 69.63 E, and elevation 69.04 m). The collected samples were separately placed in sterile polythene bags and transferred to the Laboratory of Microbial Biotechnology, Institute of Molecular Biology and Biotechnology (IMBB), The University of Lahore (UOL), Lahore, Pakistan for microbial isolation and stored at 4 °C. Bacterial isolation was carried out using the serial dilution method, with dilutions performed up to 10^−7^. The diluted samples were spread on Luria–Bertani (LB) media plates using a glass spreader and incubated at 30 ± 1 °C for 72 h [28]. The resulting bacterial growth was observed visually, and selected colonies were repeatedly sub-cultured on LB media plates until the purified bacteria cultures were obtained. The pure single colonies were preserved at –20 °C in 50% glycerol stock for further experiments [29].

### 2.2. Screening of Rhizobacterial Strains for Phosphate Solubilization

Isolated bacterial strains were screened for their ability to solubilize phosphate Pikovskaya (PVK) agar media [30]. Bacterial cultures were spot inoculated on PVK media in triplicate and incubated at 30 ± 1 °C for 7 days. After incubation, plates were observed for halo zones around bacterial colonies. Using a measuring scale, the bacterial colony and solubilization halo zone diameters were measured in millimeters. P solubilization index (PSI) and P solubilization efficiency (PSE) were calculated by using the formula described by Ahmad et al. [31]. Bacterial strains were inoculated in PVK broth to quantify available P concentration and incubated at 30 ± 1 °C for 7 days. Bacterial cells were harvested by filtering through Whatman filter paper 42, and the solubilized P concentration was determined by the colorimetric method through a spectrophotometer (Agilent Technologies, Santa Clara, CA, USA). 

### 2.3. Potassium Solubilization Assay

For K solubilization, Aleksandrov agar media was spot inoculated by bacterial strains in triplicate [32]. After incubation for seven days at 30 ± 1 °C, the plates were flooded with iodine solution to observe the clear halo zones. K solubilization index (KSI) and K solubilizing efficiency (KSE) were determined by measuring the solubilization halo zone and bacterial colony diameter by using the formula described by Setiawati and Mutmainnah [33]. The solubilized concentration by bacterial strains was determined using a supernatant of Aleksandrov broth after one week and subjected to a flame photometer. The available K concentration was determined by plotting the standard curve.

### 2.4. Zinc Solubilization Assay

The Zn solubilizing ability of rhizobacterial strains was evaluated using Bunt and Rovira agar media [34]. The Bunt and Rovira agar medium amended with zinc oxide (1% of Zn) was spot inoculated with bacterial strains and incubated for seven days at 30 ± 1 °C. The appearance of a solubilization halo zone was considered positive for Zn solubilization. Zn solubilization efficiency (ZSE) and Zn solubilization index (ZSI) were calculated by measuring the diameter of bacterial growth and solubilization halo zones of bacterial isolates by using the formula as reported by Ahmad et al. [31]. Similarly, bacterial strains were inoculated in Bunt and Rovira broth amended with zinc oxide (1% of Zn). After one week of incubation at 30 ± 1 °C, broth cultures were filtered, and available Zn concentration was determined through an atomic absorption spectrophotometer. 

### 2.5. Manganese Solubilization Assay

Mn solubilization by rhizobacterial strains was carried out in nutrient agar media supplemented with 50 mM MnO_2_ [35]. After incubation of media plates at 30 ± 1 °C for 72 h, halo zone formation around the bacterial growth was determined visually by adding iodine solution as an indicator. Mn solubilization index (MSI) and solubilization efficiency (MSE) were determined according to the formula reported by Ijaz et al. [35]. The Mn solubilized concentration was determined by inoculating nutrient broth amended with MnO_2_ with rhizobacterial strains. After one week of incubation, culture filtrate was used to assess their solubilized concentration through the atomic absorption spectrophotometer.

### 2.6. Determination of Indole Acetic Acid

The selected bacterial strains were tested for indole-3-acetic acid (IAA) production using sulfide–indole-motility (SIM) media. Test tubes were incubated at 30 ± 1 °C for 48 h and observed for the production of a red-colored ring by adding 1 mL of KOVAC’S reagent into test tubes. To quantify IAA production, bacterial strains were inoculated in nutrient broth in the presence and absence of L-tryptophan (5 mg mL^−1^) [36]. The setup was incubated in triplicate at 30 ± 1 °C and 100 rpm for 72 h. The cultures were harvested by centrifugation up to 10,000× *g*, and 1 mL of supernatant was treated with 2 mL of Salkowski reagent. The optical density of the reaction mixture and IAA working standards were read at 530 nm through a spectrophotometer. The IAA concentration was calculated by drawing the standard curve.

### 2.7. Determination of Enzymatic Activities

Bacterial strains were assessed for their protease and amylase activities using methods reported by Cappuccino and Welsh [37]. The catalase, coagulase, and urease activities were determined by following the standard procedures described in Cheesbrough [38]. For protease activity, bacterial strains were screened for their ability to produce protease enzyme on skim milk agar (SMA) media. The bacterial colony was spot-inoculated on media using a sterile needle. The appearance of halo zones around bacterial colonies was checked after 48 h of incubation at 30 ± 1 °C. The amylase activity was determined by inoculating freshly grown bacterial cultures on media containing starch agar, and plates were incubated at 30 ± 1 °C for 48 h. Iodine solution was poured on bacterial colonies for a few seconds and recorded data. The catalase activity was determined by adding a few drops of hydrogen peroxide (H_2_O_2_) into the pure bacterial cells on a glass slide using a dropper. The slide was observed for bubble formation. For coagulase activity, distilled water drop was added on a glass slide, and the bacterial colony was emulsified to make a suspension. A few drops of plasma were placed on the bacterial colony, and the slide was examined for clump formation after 10 s. Christensen’s urea slant media was used to check the urease activity of bacterial strains. The slant media was incubated at 30 ± 1 °C and examined after 72 h.

### 2.8. Morphological and Microscopic Characterization

The LB agar plates were used to examine the morphological characteristics of bacterial strains. The colonies were illustrated based on shape, size, surface, color, opacity, elevation, margin, and consistency [37]. For the triple sugar iron (TSI) test, a pure colony of bacterial strain was streaked on TSI agar media and examined after 24 h of incubation. Production of gas, blackening of media, and changes in the color of slant and butt were observed [38]. Microscopic observation was conducted to determine the bacterial isolates’ form, Gram reactivity, and motility. Vincent and Humphrey’s [36] method was used for Gram staining. A bacterial colony was taken with the help of a sterile loop and emulsified in a water drop on a glass slide. The slide was heat fixed by passing through Bunsen flame three times. Crystal violet was added to the slide and washed with running water after a minute. After the primary stain, Gram iodine was added as a mordant. The slide was then flooded with ethanol for 30 s and washed with tap water. Afterward, safranin was added and allowed to stain for one minute. The slide was washed, air-dried, and examined under a microscope to study the color and shape of the colony [36].

### 2.9. Molecular Identification of Selected Bacterial Strains

The most promising rhizobacterial strains were chosen for 16S rRNA gene sequence analysis based on the mineral’s solubilizing ability and enzymatic activities. The genomic DNA of selected strains was extracted from the cell culture using proteinase K treatment [39]. The 16S rRNA region of 2.5 µL genomic DNA was amplified through thermocycler (Eppendorf, Enfield, Connecticut, United States) using the universal forward primers 785F (5′–GGATTAGATACCCTGGTA–3′) and reverse primers 907R (5′–CCGTCAATTCMTTTRAGTTT–3′). The size of the amplified 16S rRNA region was confirmed by running gel electrophoresis through 1% agarose gel along with GeneRuler 1 kb DNA (Fermentas; Thermo Scientific, Waltham, MA, USA). The PCR product was purified through a PCR purification kit (Favorgen, Taiwan), and the 16S rRNA partial gene was sequenced from the commercial service of Macrogen Seoul, Korea (https://dna.macrogen.com/, accessed on 10 July 2022). The consensus sequence from forward and reverse sequences was obtained using the software Bioedit version 7.2 (https://bioedit.software.informer.com/7.2/, accessed on 1 September 2022). The consensus sequences were analyzed on highly similar sequences (megablast) program of nucleotides blast (blastn) in NCBI web server (https://blast.ncbi.nlm.nih.gov/Blast.cgi, accessed on 25 September 2023). The strains were identified based on the maximum homology found in the 16S ribosomal DNA sequence (bacteria and archaea) from the RNA database using the EzBiCloud web server (https://www.ezbiocloud.net/, accessed on 25 September 2023). The sequences of closely related type strains were obtained from the EzBioCloud web service (https://www.ezbiocloud.net/, accessed on 25 September 2023) using the 16S-based-ID database. Phylogenetic analyses based on the maximum likelihood method were performed with all closely related taxa using MEGA version X with kimura 2 parameter model, using 1000 bootstrap (https://www.megasoftware.net/downloads/dload_win_gui, accessed on 25 September 2023) [40]. The sequences used for bacterial identification were submitted to the NCBI GenBank database, and accession numbers were obtained.

### 2.10. Evaluation of Bacterial Strains for Promotion of Rhodes Grass Growth

The soil culture pot experiment was conducted in plastic pots to evaluate the potential of promising rhizobacterial strains on Rhodes grass growth attributes. Based on minerals solubilization, eight bacterial strains; SM2, SM4, SM5, SM7, MS1, MS2, MS4, and MS7 were selected for pot trial. The rhizobacterial cultures were freshly grown in LB broth under shaking conditions (100 rpm) at 30 ± 1 °C for 48 h. Before seed sowing, Rhodes grass seeds were soaked in bacterial cultures of 0.70 optical density at 600 nm for one hour. In the uninoculated control group, seeds were soaked in LB broth without bacterial inoculation. This experiment was performed under natural climatic conditions in IMBB, UOL, Pakistan greenhouse at 31.39 N latitude, 74.24 E longitude, and 206 m elevation. The sieved soil of 9 kg weight was added to each pot, and seeds were sown at a depth of 2 inches. Pots were arranged in a completely randomized design (CRD) with three replications for each treatment. Plants were watered on alternate days, and Hoagland solution was added once a week. After 4 weeks of seed germination, plants were harvested carefully, and data regarding root and shoot growth were recorded. Roots were washed with tap water and separated from shoots. Growth parameters like root length and shoot length were recorded through a measuring scale. Fresh and dry biomasses of roots and shoots were weighed through a top-loaded electrical weight balance.

### 2.11. Statistical Analysis

Statistical analysis was performed by using one-way analysis of variance (ANOVA) in completely randomized design (CRD) with Statistix 8.1 software to evaluate the effects of rhizobacterial strains on the growth parameters of Rhodes grass. Mean values from in vitro and in vivo assays were compared using the Tukey HSD test. At the same time, the pot experiment was analyzed through the least significant difference (LSD) test at a 5% probability level (*p* ≤ 0.05) [41]. Values presented in the tables are the mean of triplicates ± standard error (SE), which means sharing similar letters was not significantly different.

## 3. Results

A total of 25 bacterial strains were isolated from the Rhodes grass rhizosphere samples collected from two different locations, site I (Rahim Yar Khan, Punjab, Pakistan) and site II (Ghotki, Sindh, Pakistan. Of these 25 strains, 10 rhizobacterial strains were isolated from site I, and 15 rhizobacterial strains were isolated from site II.

### 3.1. Rhizobacterial Strains Demonstrated Phosphate Solubilization Potential

**Figure 1:**
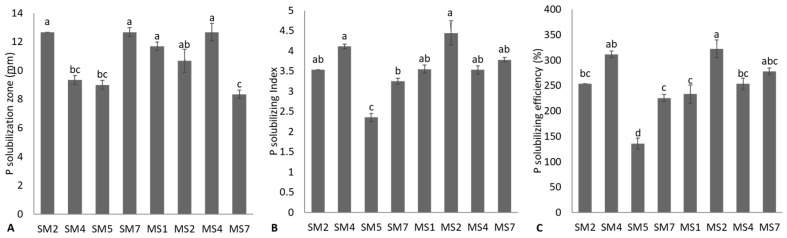
Phosphate (P) solubilization potential of bacterial strains based on P solubilization zone formation (**A**), P solubilizing index (**B**), and P solubilizing efficiency (**C**) of bacterial strains; values are mean of triplicates ± standard error and mean sharing similar letters were not significantly different from each other.

The isolated bacterial strains were assessed for their mineral solubilization potential of different insoluble minerals, such as P, K, Zn, and Mn solubilization. Out of 25 isolated strains, eight rhizobacterial strains were positive for P-solubilization coded as SM2, SM4, SM5, SM7, MS1, MS2, MS4 and MS7 (Figure 1). The maximum halo zone diameter was shown by rhizobacterial strains SM2, SM7, and MS4; these strains were statistically (*p* ≤ 0.05) similar to MS2. Strain MS7 was the lowest to report P-solubilization zone and bacterial growth zone diameters. The strains MS2 followed by SM4 strains reported the maximum PSI and PSE. These strains were statistically non-significant (*p* ≤ 0.05) with strains SM2, MS1, MS4, and MS7 in the case of PSI and with strain MS7 in the case of PSE. The strain SM5 was the lowest to report PSI and PSE. The quantitative P solubilization and reduction in pH were observed, and data are given in Table 1. Strain MS2 reported a maximum available P concentration of 27.45 ± 0.14 ppm and was statistically non-significant (*p* ≤ 0.05) to strains SM2 and SM4. The increase in available P concentration was observed due to the reduction in pH of the PVK medium. The highest pH reduction in the PVK medium was found by strain SM2 (4.24 ± 0.02) and SM4 (4.39 ± 0.03).

### 3.2. Rhizobacterial Strains Demonstrated Zinc Solubilization Potential

**Figure 2:**
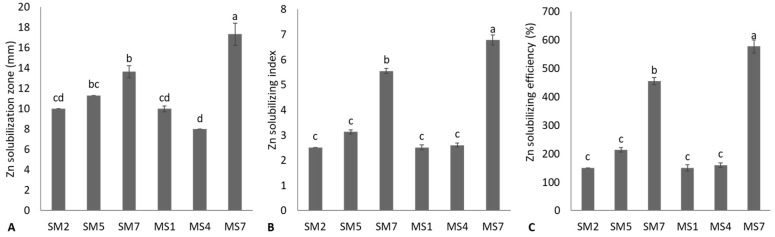
Zinc (Zn) solubilization potential of bacterial strains based on Zn solubilization zone formation (**A**), Zn solubilizing index (**B**), and Zn solubilizing efficiency (**C**) of bacterial strains; values are mean of triplicates ± standard error and mean sharing similar letters were not significantly different from each other.

These P-solubilizing bacterial strains were tested for Zn-solubilization; six rhizobacterial strains were positive (Figure 2). Strain MS7, followed by SM7, showed the maximum Zn-solubilization zone diameter. The MS4 reported a minimum Zn-solubilization diameter and was statistically (*p* ≤ 0.05) similar to MSI and SM2. The strains MS7 and SM7 reported the highest ZSI and ZSE. Strains SM2, SM5, MSI, and MS4 reported the lowest and non-significant (*p* ≤ 0.05) ZSI and ZSE. The quantitative Zn solubilization and reduction in pH were observed, and data are given in Table 2. The highest available Zn was obtained from strain MS7 (21.72 ± 0.39 ppm) due to the highest reduction in pH (4.60 ± 0.08) of medium containing insoluble Zn. After that, strain SM7 showed a better increase in available Zn (19.81 ± 0.33 ppm) due to a reduced pH (4.79 ± 0.08) of the medium containing insoluble Zn. 

### 3.3. Rhizobacterial Strains Demonstrated Manganese and Potassium Solubilization Potential

**Figure 3:**
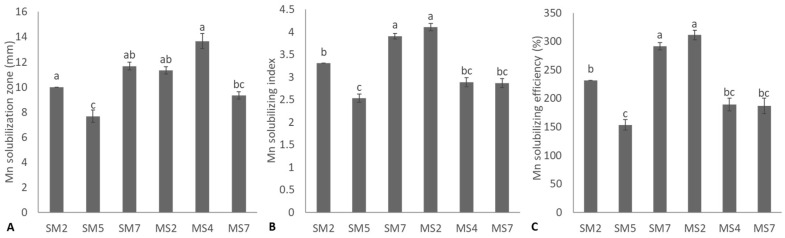
Manganese (Mn) solubilization potential of bacterial strains based on Mn solubilization zone formation (**A**), Mn solubilizing index (**B**), and Mn solubilizing efficiency (**C**) of bacterial strains; values are mean of triplicates ± standard error and mean sharing similar letters were not significantly different from each other.

**Figure 4:**
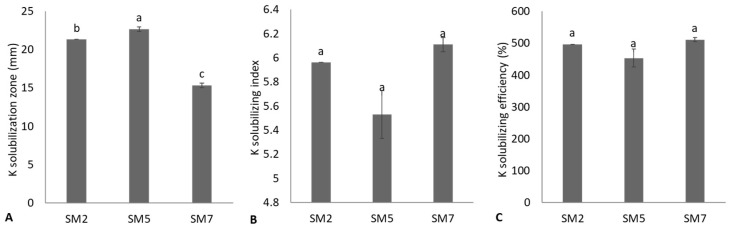
Potassium solubilization potential of bacterial strains based on potassium solubilization zone formation (**A**), potassium solubilizing index (**B**), and potassium solubilizing efficiency (**C**) of bacterial strains; values are mean of triplicates ± standard error and mean sharing similar letters were not significantly different from each other.

Six rhizobacterial strains were positive for Mn solubilization (Figure 3). Strain MS4 showed the maximum Mn solubilization zone diameter and was statistically (*p* ≤ 0.05) similar to strains SM2, SM7, MS2, and MS4. The strains SM7 and MS2 reported the highest MSI and MSE and were statistically (*p* ≤ 0.05) identical. The minimum Mn solubilization zone diameter, MSI, and MSE were observed from strain SM5. Three rhizobacterial strains (SM2, SM5, SM7) were positive for K-solubilization (Figure 4). Strain SM5 showed maximum halo zone diameter, while strain SM7 reported the lowest K-solubilization diameter. However, SM7 reported maximum KSI and KSE and was non-significant (*p* ≤ 0.05) to SM2 and SM5. Strain MS2 reported maximum available Mn concentration (11.07 ± 0.13 ppm) with the highest reduction in pH (4.12 ± 0.04) of the nutrient broth containing insoluble Mn (Table 3). The maximum available K concentration (21.6 ± 0.61 ppm) was obtained from SM4 due to the highest reduction in pH (4.60 ± 0.02) of medium containing mica (Table 3).

### 3.4. IAA Production by Rhizobacterial Strains

The rhizobacterial strains produced IAA in a medium without L-tryptophan; however, the bacterial ability for IAA production was more prominent in the presence of L-tryptophan (Table 4). Strain SM4 reported the highest production of IAA (26.87 ± 3.14 µg mL^−1^), followed by strains SM5 (22.39 ± 1.55 µg mL^−1^) and SM7 (23.74 ± 2.18 µg mL^−1^) in the presences of L-tryptophan. In the absence of L-tryptophan, strain SM4 also reported maximum IAA production (5.76 ± 1.61 µg mL^−1^) and was statistically (*p* ≤ 0.05) similar to strains SM2, SM5, and SM7.

### 3.5. Enzymatic Activities by Rhizobacterial Strains

Enzymatic activities of rhizobacterial strains include catalase, coagulase, urease, amylase, and protease activities are presented in Table 5. All tested rhizobacterial strains showed catalase production. Half of the tested strains were positive for urease activity in terms of change in color from yellow to pink. Only two rhizobacterial strains, SM7 and MS7, were positive for the coagulase activity. Comparatively, the other six rhizobacterial strains were coagulase-negative, as clumping in blood plasma was absent. Five rhizobacterial strains were protease-positive, while six were amylase-positive. The urease, protease, and amylase experiment results can be viewed in Figures S1–S3. The TSI results are also provided in Table S1 and Figure S4. 

### 3.6. Morphological Characteristics of Rhizobacterial Strains

The morphological characteristics of eight tested mineral solubilizing rhizobacterial strains varied widely (Table 6). These bacterial colonies have circular shapes except strain SM5, which showed an irregular shape. Most colonies were small; however, strains SM4, SM5, and MS4 showed medium colony sizes. Bacterial colonies have different colors: off-white (SM5 and MS1); pure white (SM7 and MS4); skin (SM2 and MS7); yellow (SM2); and mustard (MS2). They also showed variable colony surfaces of smooth (SM2, SM4, SM78, MS7), shiny (MS5 and MS2), dull (MS1), and wrinkled (MS4). More than 62% of colonies were translucent, and 38% were opaque, while flat elevation colonies were observed in 50% of strains, and the rest were of raised and umbonate surface elevation. Most strain colonies were of wavy margin (75%), and the rest were even margin (25%). Their consistency was butyrous (62.5%), dry (12.5%), and hard (25%). All the tested strains were of Gram-positive nature (Table 7). The microscopic observation showed that seven bacterial isolates were rod-shaped, and one was cocci. Six strains were motile, and two strains were non-motile.

### 3.7. Effect of Rhizobacterial Strains on Growth Promotion of Rhodes Grass

**Figure 5:**
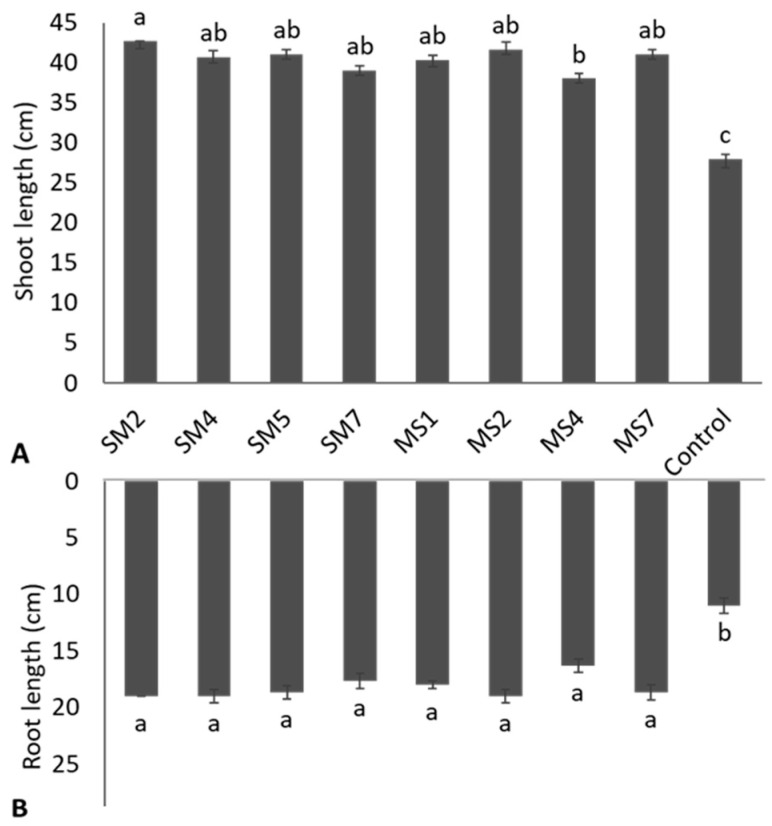
Effect of bacterial strains on shoot length (**A**) and root length (**B**) on seedlings of Rhodes grass. Values are mean of triplicates ± standard error and mean sharing similar letters were not significantly different from each other.

Bacterial inoculation significantly (*p* ≤ 0.05) enhanced Rhodes grass growth parameters regarding root length, shoot length, and fresh and dry biomasses of root and shoot (Figure S5). The shoot length and root length due to bacterial inoculation were non-significant (*p* ≤ 0.05) to each other but were statistically significant (*p* ≤ 0.05) to the uninoculated control (Figure 5). The strain SM2, followed by MS2, SM5, and MS7, reported the highest increase of 52.4%, 48.8%, 46.4%, and 46.4%, respectively, in shoot length over the uninoculated control (Figure 5A). The maximum gain of 72.7% in the root length of Rhodes grass was observed due to strains SM2, SM4, and MS2 application (Figure 5B).

**Figure 6:**
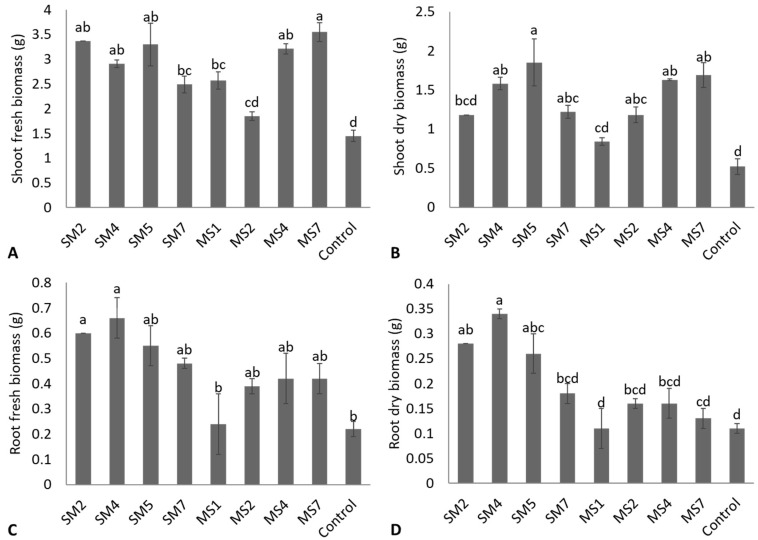
Effect of bacterial strains on shoot fresh biomass (**A**), shoot dry biomass (**B**), root fresh biomass (**C**), and root dry biomass (**D**) on seedlings of Rhodes grass. Values are mean of triplicates ± standard error and mean sharing similar letters were not significantly different from each other.

Strain MS7 reported the maximum increase of 144.8% in shoot fresh biomass and was statistically non-significant (*p* ≤ 0.05) to strains SM4, SM5, and MS4; however, these strains were statistically (*p* ≤ 0.05) different from the uninoculated control (Figure 6A). The lowest increase of 27.6% was obtained from strain MS2 and was statistically non-significant (*p* ≤ 0.05) to the uninoculated control. The highest shoot dry biomass was reported by strain SM5, with an increase of 255.8% over the uninoculated control (Figure 6B). The shoot dry biomass due to strain SM5 was statistically non-significant (*p* ≤ 0.05) to strains SM4, SM7, MS2, MS4, and MS7; however, these strains showed statistically (*p* ≤ 0.05) highest shoot dry biomass over uninoculated control. The increase in root fresh biomass due to applied bacterial strains was statistically non-significant (*p* ≤ 0.05) to uninoculated except strains SM2 and SM4, which reported the maximum increase of 172.7% and 200% in root fresh biomass (Figure 6C). The bacterial strains also reported an increase in root dry biomass; however, it was statistically non-significant (*p* ≤ 0.05) to the uninoculated control except SM2, SM4, and SM5 (Figure 6D). The highest gain of 209% was reported by strains SM4, over uninoculated control, and was statistically (*p* ≤ 0.05) similar to SM2 and SM5. The uninoculated control showed minimum root length, shoot length, shoot and root fresh and dry biomass (Figure 5 and Figure 6).

### 3.8. Identification of Rhizobacterial Strains

**Figure 7:**
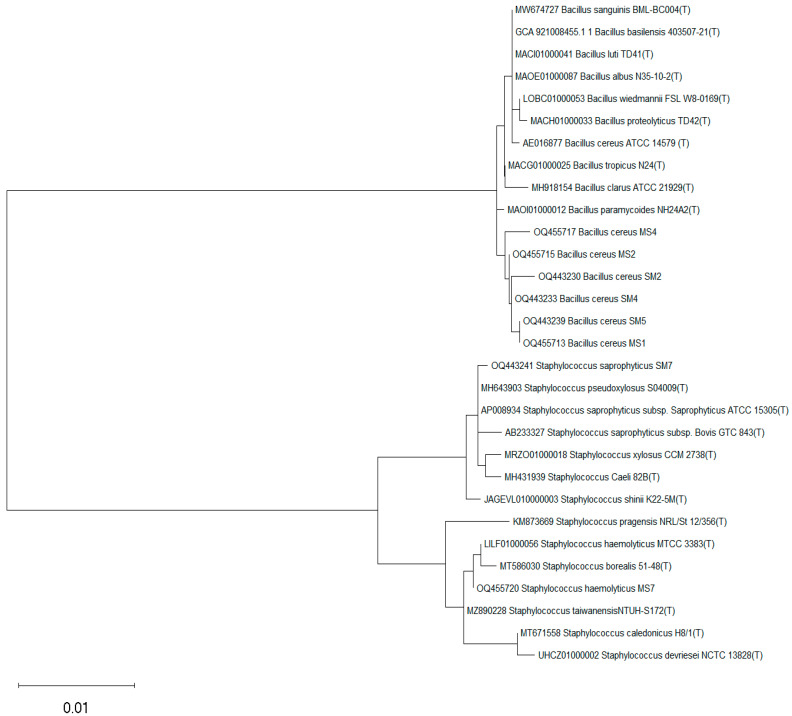
The phylogenetic tree of strains with their closet matching strains; SM2 (OQ443230), SM4 (OQ443233), SM5 (OQ443239), MS1 (OQ455713), MS2 (OQ455715), and MS4 (OQ455717) were identified as *Bacillus cereus*, while strains SM7 (OQ443241) and MS7 (OQ455720) were identified as *Staphylococcus saprophyticus* and *Staphylococcus haemolyticus*, respectively.

The rhizobacterial strains SM2, SM4, SM5, MS1, MS2, and MS4 were identified as *Bacillus cereus*, while strains SM7 and MS7 were identified as *Staphylococcus saprophyticus* and *Staphylococcus haemolyticus*, respectively through 16S rRNA gene sequencing (Table 8). The sequences of identified strains were submitted in the gene bank of NCBI, and obtained accession numbers are given in Table 8. The retrieved sequences were analyzed using different bioinformatics tools such as DNA Star, BLAST, and MEGA X. All the strains showed an excellent blend of microflora belonging to different bacterial species. The neighbor-joining method was used to construct the phylogenetic tree. The phylogenetic tree of identified *Bacillus cereus* strains SM2 (OQ443230), SM4 (OQ443233), SM5 (OQ443239), MS1 (OQ455713), MS2 (OQ455715), and MS4 (OQ455717), while, SM7 (OQ443241) and MS7 (OQ455720) were identified as *Staphylococcus saprophyticus* and *Staphylococcus haemolyticus* with their closest matching strains (Figure 7).

## 4. Discussion

Plants often face a significant limitation in mineral availability, as minerals exist in insoluble forms that plants cannot take up despite their importance in proper plant growth and functioning. Plant growth-promoting bacteria offer a promising alternative to traditional agrochemicals, as they enhance the bioavailability of nutrients in the soil and encourage plant growth and development [42,43,44]. Applying rhizobacteria as biofertilizers can improve plant growth and yield and is crucial for maintaining sustainable agriculture and soil fertility [45]. Therefore, the present study aimed to isolate mineral solubilizing bacteria from the rhizosphere soil of Rhodes grass and evaluate their potential on plant growth attributes. The selected strains were identified as *Bacillus* and *Staphylococcus* spp. through 16S rRNA gene sequence analysis. A limited number of studies reported the rhizobacterial strain’s interaction with Rhodes grass. Gupta et al. [46] reported the highest abundance of diverse diazotroph communities containing the *NifH* gene in the plant’s stem and root parts. 

Among tested bacterial strains, eight bacterial strains showed strong P-solubilization in solid and liquid mediums. Rhizobacterial strains *B. cereus* MS2, *B. cereus* SM4, and *S. haemolyticus* MS7 reported the highest P solubilizing index and efficiency, available P concentration, and maximum reduction in pH of the medium. Similarly, Zaheer et al. [47] reported P solubilization by *Pseudomonas* sp. AZ5 and *Bacillus* sp. AZ17. They reported an increase in plant growth, yield, and nutrient uptake in chickpeas through the application of *Pseudomonas* sp. AZ5 and *Bacillus* sp. AZ17 [47]. Ahmad et al. [48] also reported predominant P solubilization by *Bacillus subtilis* IA6, *Paenibacillus polymyxa* IA7, *Bacillus* sp. IA16, and *Bacillus aryabhattai* IA20. These strains also demonstrated their potential to promote cotton growth under nutrient-depleted conditions [48]. Numerous studies have also reported similar P solubilization by diverse microorganisms [49,50,51,52,53]. Such bacterial strain uses various strategies to solubilize insoluble phosphate, which might be the production of low molecular mass organic acid, including acetic, butyric, citric, fumaric, gluconic, glucuronic, lactic, maleic, malic, oxalic, propionic, succinic, tartaric, and valeric acids [49,51,54]. These organic are produced by many bacterial strains in the natural environment and in vitro conditions and play an important role in the solubilization of phosphate through chelating mineral ions and reducing the pH of the medium [50,52,55].

In the current study, six rhizobacterial strains demonstrated qualitative and quantitative Zn solubilization. The maximum Zn solubilization index, Zn solubilization efficiency, available Zn concentration, and reduction in pH of the medium were obtained from strains *S. saprophyticus* SM7 and *S. haemolyticus* MS7. Previously, similar findings were reported in various studies [56,57,58,59,60,61]. Mumtaz et al. [57] isolated thirteen rhizobacterial strains and reported Zn and phosphate solubilization by promising *Bacillus* strains, including *Bacillus* sp. (ZM20), *B. aryabhattai* ZM31, and *B*. *subtilis* (ZM63). Similarly, Bhatt and Maheshwari [59] reported Zn solubilization by *B. megaterium* CDK25 through phytase activities. Such bacterial strains might be accomplished by various mechanisms for Zn solubilization, like proton extrusion, production of organic acids, and chelating agents [62,63,64,65]. In the current study, six bacterial strains showed their potential to solubilize Mn on nutrient agar media amended with insoluble MnO_2,_ while only three rhizobacterial strains were positive for K solubilization. Strains *B. cereus* MS2 and *S. haemolyticus* MS7 reported maximum qualitative and quantitative Mn solubilization, while *S. saprophyticus* SM7 reported highest qualitative and quantitative K solubilization. Previously, Mn solubilization by microbial strains was reported by various researchers [36,66]. Solubilization of Mn and K insoluble minerals by rhizobacterial strains may involve multiple mechanisms, including organic acid production and protons by acidolysis [67,68]. The primary process for mineral solubilization by these rhizobacterial strains might be due to organic acid production, which reduces the soil pH and solubilizes minerals [66,69]. These organic acids include 2-keto gluconic, gluconic, butyric, lactic, glyoxylic, malic, fumaric, propionic, pyruvic, succinic, tartaric, citric, fumaric, and acetic acids [21,47,70,71,72,73,74,75]. Most rhizobacterial strains tested positive for enzymatic activities such as catalase, coagulase, urease, protease, and amylase. These enzymes could play an important role in plant growth promotion by acting as biocontrol agents against various plant pathogens and affecting characteristics of biochemical processes [76].

The tested rhizobacterial strains in the present study demonstrated the production of IAA in the presence and absence of L-tryptophan. These strains showed significant variation in IAA production, which could be due to the utilization ability of L-tryptophan by various rhizobacterial strains. Such variation in IAA production by bacterial strains was also reported by many authors [77,78,79,80]. In the current study, strains *B. cereus* SM2, *B. cereus* MS4, *B. cereus* SM5, and *S. haemolyticus* MS7 yielded the highest IAA production in the presence and absence of L-tryptophan. Similarly, Mumtaz et al. [57] reported the production of IAA by multiple trait mineral solubilizing *Bacillus* strains ZM20, ZM27, ZM63, and S10, both in the presence and absence of L-tryptophan. In the presence of L-tryptophan, IAA production was boosted by rhizobacterial strains. Batista et al. [81] demonstrated the complete set of genomes of *B. thuringiensis* RZ2MS9 required in indole-3-pyruvate and tryptamine pathways for IAA production. The L-tryptophan is a precursor of IAA and stimulates its production by bacteria and responds as a phytostimulatory effect in plants [82]. Our previous findings reported an increase in plant growth due to the production of IAA, especially in the presence of L-tryptophan [31,35,57,72,83]. In the current study, the inoculation with rhizobacterial strains promoted Rhodes grass seedlings, which could be due to phytostimulation of IAA. Under natural soil conditions, bacteria can utilize L-tryptophan released from root exudates and degrading roots, and the auxin biosynthesis in the rhizosphere is promoted [84]. Although IAA production was highest in all tested strains in the presence of L-tryptophan; however, strain MS1 showed the lowest IAA concentration up to 7.38 ± 0.36 µg mL^−1,^ which might be due to the activity of IAA oxidase and peroxidase activities that degrade auxin [85]. 

The inoculation of Rhodes grass seedlings with bacterial strains significantly promoted plant growth attributes such as shoot length, root length, root and shoot dry, and fresh biomasses over the uninoculated control. The present study’s findings are consistent with previously reported studies [31,86,87,88,89,90,91]. Ahmad et al. [48] reported that cotton seeds inoculated with *Bacillus* strains having the ability to solubilize P and Zn significantly promoted growth attributes over uninoculated control. PGPRs such as *Staphylococcus pasteuri*, *Bacillus cereus*, and *Bacillus velezensis* have been reported in earlier studies as promising mineral solubilizers with multifarious plant growth-promoting activities [92,93,94]. Among these plant growth activities, production of IAA, siderophores, biofilm formation, and ACC-deaminase activities play important roles in promoting plant growth, which might be true in the current study. An increase in growth parameters could be associated with the potential of bacterial strains to perform various enzymatic activities, solubilize insoluble minerals, including P, Zn, K, and Mn, and increase their uptake plants. These beneficial rhizobacterial strains could enhance nutrient availability and promote their uptake under nutrient-deficient soils [95]. Such bacterial strains are economically and ecologically more significant and help to overcome mineral deficiency. Inoculation with such mineral-solubilizing bacterial strains could enhance nutrient availability and significantly promote Rhodes grass growth attributes.

## 5. Conclusions

The present study concluded that the *Bacillus* and *Staphylococcus* strains demonstrated in vitro mineral solubilization, enzymatic activities, and IAA production. Among tested strains, *B. cereus* SM4, *S. saprophyticus* SM7, *B. cereus* MS2, and *S. haemolyticus* MS7 demonstrated maximum solubilization of insoluble phosphate, zinc, manganese, and potassium minerals. Strain *B. cereus* SM2 and *B. cereus* SM4 reported maximum production of IAA in the presence and absence of its precursor. The inoculation of Rhodes grass with tested rhizobacterial strains significantly promoted seedling growth. Maximum shoot growth was obtained from inoculation with *B. cereus* SM5 and *S. haemolyticus* MS7, while maximum root growth was reported by strains *B. cereus* SM2 and *B. cereus* SM4. These strains could be attractive bioinoculants to enhance Rhodes grass production because they possess multiple PGP attributes and enzymatic activities and can address the problem of nutrient deficiencies. These strains could be more efficient and eco-friendly alternatives to chemical fertilizers, leading to a promising approach to sustainable agriculture.

## Figures and Tables

**Table 1:** The available concentration of P and reduction in pH of the media by rhizobacterial strains in response to insoluble tricalcium phosphate.

Bacterial Strains	Available P Concentration (ppm)	pH Reduction in Response to Phosphate
Control	3.05 ± 0.34 ^e^	6.87 ± 0.06 ^a^
SM2	26.00 ± 0.65 ^abc^	4.51 ± 0.15 ^de^
SM4	26.60 ± 0.15 ^ab^	4.39 ± 0.03 ^ef^
SM5	24.35 ± 0.41 ^c^	4.82 ± 0.06 ^c^
SM7	20.65 ± 0.25 ^d^	5.21 ± 0.02 ^b^
MS1	24.85 ± 0.40 ^c^	4.65 ± 0.02 ^cd^
MS2	27.45 ± 0.14 ^a^	4.24 ± 0.02 ^f^
MS4	25.45 ± 0.55 ^bc^	4.58 ± 0.14 ^de^
MS7	22.30 ± 0.61 ^d^	4.93 ± 0.05 ^b^
CVC	1.7496	0.2077

CVC stands for critical value for comparison at *p* ≤ 0.05; The means sharing the same alphabet letters were non-significant from each other.

**Table 2:** The available concentration of Zn and reduction in pH of the media by rhizobacterial strains in response to zinc oxide.

Bacterial Strains	Available Zn Concentration (ppm)	pH Reduction in Response to Zinc Oxide
Control	2.16 ± 0.09 ^f^	6.78 ± 0.01 ^a^
SM2	19.11 ± 0.29 ^bc^	5.32 ± 0.09 ^c^
SM4	NT	NT
SM5	17.96 ± 0.93 ^c^	5.18 ± 0.06 ^cd^
SM7	19.81 ± 0.33 ^b^	4.79 ± 0.08 ^de^
MS1	11.63 ± 0.29 ^e^	6.17 ± 0.4 ^b^
MS2	15.33 ± 0.45 ^d^	5.01 ± 0.02 ^cd^
MS4	NT	NT
MS7	21.72 ± 0.39 ^a^	4.60 ± 0.08 ^e^
CVC	1.5441	0.2258

The symbol NT stands for not tested; CVC stands for critical value for comparison at *p* ≤ 0.05; The means sharing the same alphabet letters were non-significant from each other.

**Table 3:** The available concentration of Mn and K and reduction in pH of the media by rhizobacterial strains in response to their insoluble sources.

Bacterial Strains	Available Mn Concentration (ppm)	pH Reduction in Response to Manganese Oxide	Available K Concentration (ppm)	pH Reduction in Response to Zinc Oxide
Control	3.01 ± 0.06 ^f^	6.90 ± 0.02 ^a^	2.99 ± 0.29 ^d^	6.92 ± 0.06 ^a^
SM2	7.65 ± 0.29 ^b^	4.45 ± 0.04 ^c^	21.6 ± 0.61 ^a^	4.60 ± 0.02 ^d^
SM4	NT	NT	NT	NT
SM5	4.79 ± 0.13 ^e^	4.80 ± 0.03 ^b^	17.86 ± 0.72 ^b^	5.18 ± 0.02 ^c^
SM7	5.15 ± 0.14 ^d^	5.08 ± 0.05 ^b^	11.54 ± 0.38 ^c^	6.18 ± 0.03 ^b^
MS1	NT	NT	NT	NT
MS2	11.07 ± 0.13 ^a^	4.12 ± 0.04 ^d^	NT	NT
MS4	6.86 ± 0.14 ^c^	4.69 ± 0.04 ^b^	NT	NT
MS7	9.87 ± 0.35 ^ab^	4.39 ± 0.03 ^c^	NT	NT
CVC	0.3154	0.2989	2.3377	0.2110

The symbol NT stands for not tested; CVC stands for critical value for comparison at *p* ≤ 0.05; The means sharing the same alphabet letters were non-significant from each other.

**Table 4:** Indole acetic acid (IAA) production by bacterial strains in the presence and absence of L-tryptophan.

Bacterial Strains	IAA Production without L-Tryptophan (µg mL^−1^)	IAA Production with L-Tryptophan (µg mL^−1^)
SM2	5.12 ± 1.91 ^ab^	17.89 ± 2.51 ^c^
SM4	5.76 ± 1.61 ^a^	26.87 ± 3.14 ^a^
SM5	4.96 ± 0.43 ^ab^	22.39 ± 1.55 ^b^
SM7	3.60 ± 1.66 ^bcd^	13.94 ± 1.40 ^de^
MS1	2.37 ± 1.71 ^d^	7.38 ± 0.36 ^f^
MS2	2.92 ± 1.86 ^cd^	12.48 ± 1.75 ^e^
MS4	4.07 ± 1.09 ^bc^	15.87 ± 2.90 ^cd^
MS7	5.02 ± 0.55 ^ab^	23.74 ± 2.18 ^b^
CVC	1.6539	2.5692

CVC stands for critical value for comparison at *p* ≤ 0.05; The means sharing the same alphabet letters were non-significant from each other.

**Table 5:** Enzymatic activities of rhizobacterial strains.

Bacterial Strains	Urease Activity	Catalase Activity	Protease Activity	Coagulase Activity	Amylase Activity
SM2	−ve	+ve	+ve	−ve	+ve
SM4	+ve	+ve	−ve	−ve	−ve
SM5	−ve	+ve	+ve	−ve	+ve
SM7	+ve	+ve	−ve	+ve	+ve
MS1	−ve	+ve	+ve	−ve	+ve
MS2	+ve	+ve	−ve	−ve	+ve
MS4	−ve	+ve	+ve	−ve	+ve
MS7	+ve	+ve	+ve	+ve	−ve

+ve indicates the trait’s presence, and −ve shows the absence of the trait.

**Table 6:** Colony characteristics of bacterial strains.

Strains	Shape	Size	Surface	Color	Opacity	Elevation	Margin	Consistency
SM2	Circular	Small	Smooth	Skin	Opaque	Flat	Even	Butyrous
SM4	Circular	Medium	Smooth	Yellow	Opaque	Raised	Even	Butyrous
SM5	Irregular	Medium	Shiny	Off white	Translucent	Raised	Wavy	Butyrous
SM7	Circular	Small	Smooth	Pure white	Translucent	Flat	Even	Butyrous
MS1	Circular	Small	Dull	Off white	Translucent	Flat	Even	Dry
MS2	Circular	Small	Shiny	Mustard	Translucent	Raised	Even	Butyrous
MS4	Circular	Medium	Wrinkle	Pure white	Opaque	Umbonate	Wavy	Hard
MS7	Circular	Small	Smooth	Skin	Translucent	Flat	Even	Hard

**Table 7:** Microscopic observation of mineral solubilizing bacteria.

Strains	Form	Gram Reaction	Color	Motility
SM2	Rod	Gram +ve	Purple	Motile
SM4	Rod	Gram +ve	Purple	Motile
SM5	Rod	Gram +ve	Purple	Motile
SM7	Cocci	Gram +ve	Purple	Non-motile
MS1	Rod	Gram +ve	Purple	Motile
MS2	Rod	Gram +ve	Purple	Motile
MS4	Rod	Gram +ve	Purple	Motile
MS7	Cocci	Gram +ve	Purple	Non-motile

**Table 8:** Identification of bacterial strains based on their 16S rRNA sequencing.

Strains	Identification by 16S rRNA Gene Sequencing	Similarity with Type Strains (%)	Size (bp)	Accession No.
SM2	*Bacillus cereus* ATCC 14579^T^ (AE016877)	99.66	1466	OQ443230
SM4	*Bacillus cereus* ATCC 14579^T^ (AE016877)	99.86	1486	OQ443233
SM5	*Bacillus cereus* ATCC 14579^T^ (AE016877)	99.86	1501	OQ443239
SM7	*Staphylococcus saprophyticus* subsp. *Saprophyticus* ATCC 15305^T^ (AP008934)	99.93	1512	OQ443241
MS1	*Bacillus cereus* ATCC 14579^T^ (AE016877)	99.86	1463	OQ455713
MS2	*Bacillus cereus* ATCC 14579^T^ (AE016877)	99.86	1475	OQ455715
MS4	*Bacillus cereus* ATCC 14579^T^ (AE016877)	99.86	1479	OQ455717
MS7	*Staphylococcus haemolyticus* MTCC3383^T^ (LILF01000056)	99.93	1544	OQ455720

## Data Availability

The partial gene sequences of *Bacillus cereus* strains SM2 (OQ443230), SM4 (OQ443233), SM5 (OQ443239), MS1 (OQ455713), MS2 (OQ455715), and MS4 (OQ455717), *Staphylococcus saprophyticus* strain SM7 (OQ443241) and *Staphylococcus haemolyticus* strain MS7 (OQ455720) can be availed using Gen Bank Accession Numbers, which were deposited in the NCBI database.

## Supplementary Materials

The following supporting information can be downloaded at https://www.mdpi.com/article/10.3390/microorganisms11102543/s1, Figure S1: Urease activity of rhizobacterial strains; Figure S2. Protease activity of rhizobacterial strains; Figure S3. Amylase activity of rhizobacterial bacteria; Figure S4. View of triple sugar iron due to inoculation with rhizobacterial strains; Figure S5. Pictorial view of a pot experiment conducted to evaluate the effect of various bacterial strains on seedlings of Rhodes grass. Table S1. The results of the triple sugar iron test of rhizobacterial strains from Rhodes grass rhizosphere.
