# Supplementary material for: Mineral Solubilizing Rhizobacterial Strains Mediated Biostimulation of Rhodes Grass Seedlings
Source: Microorganisms. 2023 Oct 12;11(10):2543. doi: 10.3390/microorganisms11102543 (PMC10609362; doi:10.3390/microorganisms11102543)
Supplement: Supplementary file 1 [file microorganisms-11-02543-s001.zip › microorganisms-2614027-supplementary.pdf]

microorganisms

MDPI

Article

Mineral Solubilizing Rhizobacterial Strains Mediated Biostimulation of Rhodes Grass Seedlings

Saira Mushtaq [1], Maha Afzal [1], Uzma Mushtaq [1], Muhammad Zahid Mumtaz [1,*], Ghulam Rasool [1], Hayssam M. Ali [2], Tahir Naqqash [3], Muhammad Fakhar-U-Zaman Akhtar [4], Ghulam Abbas [5], Lingling Li [6,7], and Shaista Javaid [1,*]

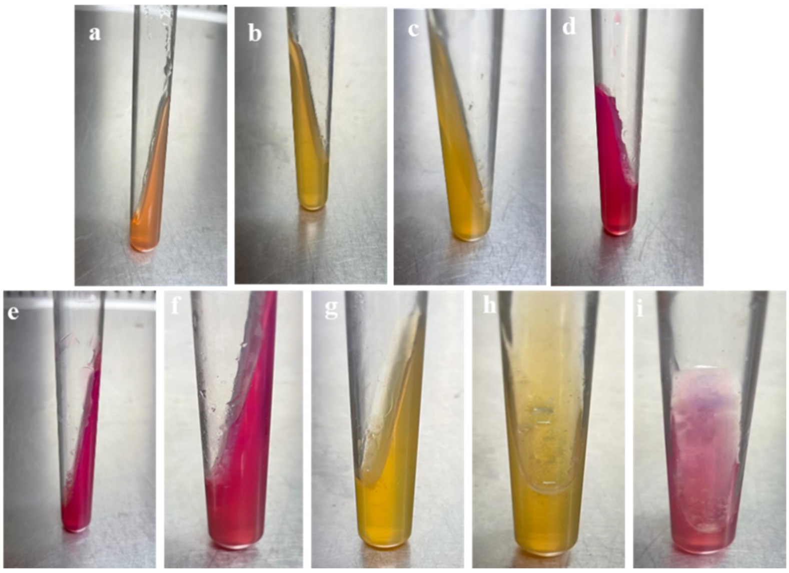

Figure S1: Urease activity of rhizobacterial strains (from left to right; (a) control; (b) SM5; (c) SM2; (d) SM7; (e) SM4; (f) MS2; (g) MS1; (h) MS4; (i) MS7).

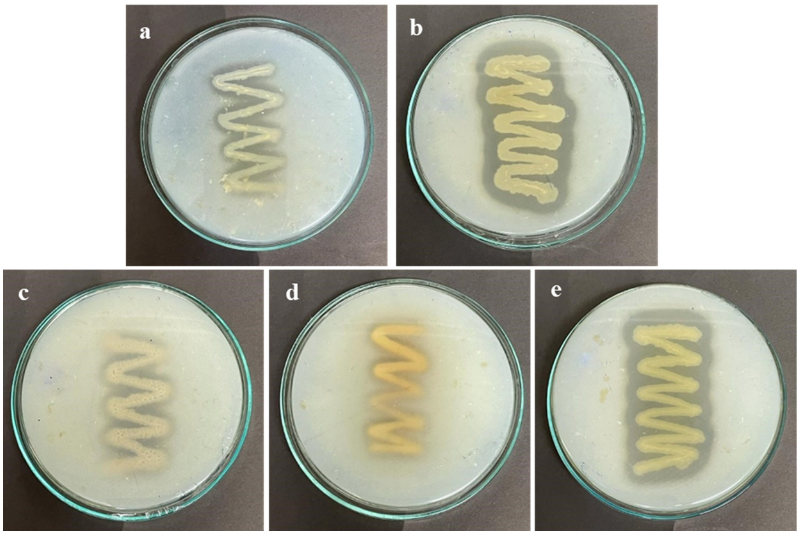

Figure S2. Protease activity of rhizobacterial strains [(a) SM2; (b) SM5; (c) MS4; (d) MS7; (e) MS1].

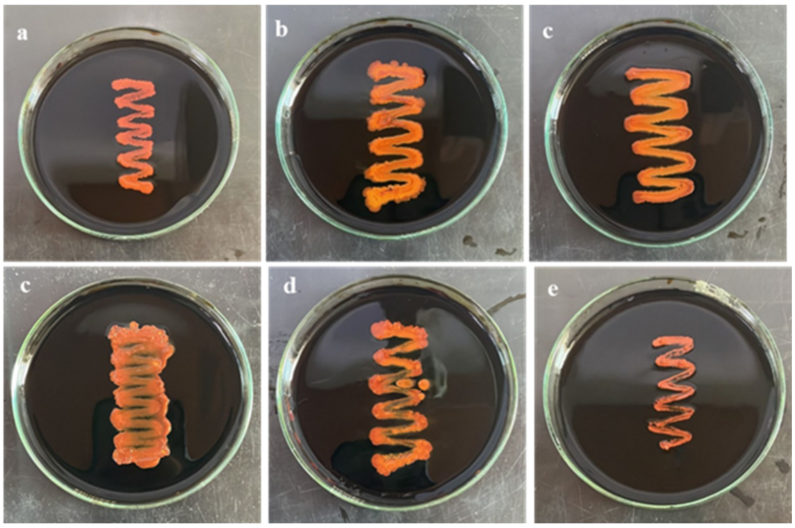

Figure S3. Amylase activity of rhizobacterial bacteria [(a) SM2; (b) SM5; (c) SM7; (d) MS1; (e) MS4; (f) MS7].

Table S1. The results of the triple sugar iron test of rhizobacterial strains from Rhodes grass rhizosphere

**Strains**	**Slant/Butt**	**Interpretation**	**Symbol**
SM2	Red Slant/ Yellow Butt	Only glucose fermentation; Peptone catabolized	K/A
SM4	Yellow Slant/ Yellow Butt	Glucose and& lactose or sucrose fermentation	A/A
SM5	Yellow Slant/ Black Butt	H_2S produced	A/ H_2S
SM7	Red Slant/ Yellow Butt	Only glucose fermentation; Peptone catabolized	K/A
MS1	Yellow Slant/ Yellow Butt	Glucose and& lactose or sucrose fermentation	A/A
MS2	Red Slant/ Yellow Butt	Only glucose fermentation; Peptone catabolized	K/A
MS4	Yellow Slant/ Yellow Butt	Glucose and& lactose or sucrose fermentation	A/A
MS7	Yellow Slant/ Yellow Butt	Glucose, sucrose fermentation and& lactose	A/A

K: Alkaline reaction; A: Acid production; H_2S: Sulfur reduction

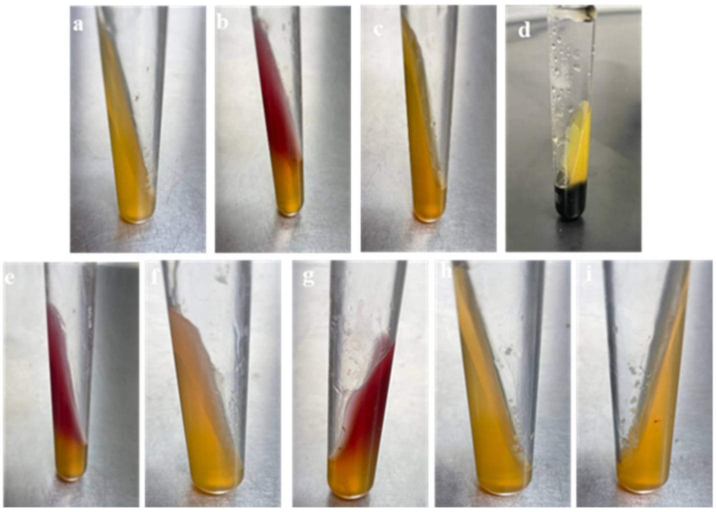

Figure S4. View of triple sugar iron due to inoculation with rhizobacterial strains [(a) Control; (b) SM2; (c) SM4; (d) SM5; (e) SM7; (f) MS7; (g) MS2; (h) MS1; (i) MS4].

Triple sugar iron test

This test was used to evaluate the ability of rhizobacterial strains to produce hydrogen sulfide (H_2S) and ferment sugars such as glucose, sucrose, and lactose. Results revealed that all the rhizobacterial strains ~~variation~~varied to ferment sugar (Table S1 and Figure S4). The butt color indicates the fermentation of glucose. In contrast, the slant color indicates sucrose and lactose fermentation. H_2S gas production results in the blackening of the medium.

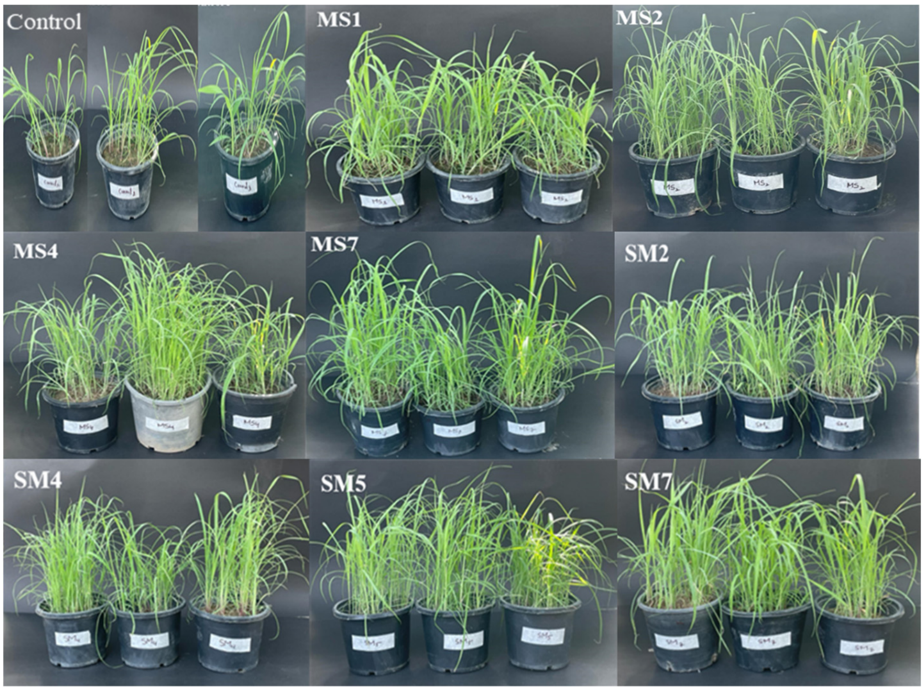

Figure S5. Pictorial view of a pot experiment conducted to evaluate the effect of various bacterial strains on seedlings of Rhodes grass.
